# Low-Power Holmium Laser Therapy for Urethral Strictures at Ninh Thuan Province General Hospital, Vietnam

**DOI:** 10.2174/0118715303389695251030101747

**Published:** 2026-01-27

**Authors:** Thach Le-Huy, Thắng Lê Quốc, Phuong Dang Quoc, Thien Dong Xuan, Truyen Bao Dai, Trang Tu Xuan, Linh Truong Thi My, Francesco Inchingolo, Kieu C.D. Nguyen, Luigi Santacroce, Ciro Gargiulo-Isacco

**Affiliations:** 1 Ninh Thuan General Hospital, Phan Rang-Tháp Chàm, Ninh Thuan Province, 59000, Vietnam;; 2 Department of Interdisciplinary Medicine, School of Medicine, University of Bari “Aldo Moro”, Bari 70124, Italy

**Keywords:** Holmium:YAG, internal urethrotomy, laser, urinary tract infection (UTI), urethral stent (JJ stent), HPV

## Abstract

**Introduction:**

Urethral stricture is a common urological disease characterized by a narrowing of the urethra leading to functional changes that reduce or completely block urine flow from the kidney to the bladder. This condition significantly affects the patient’s quality of life and can lead to serious complications, such as urethral dilatation and hydronephrosis, which may result in irreversible kidney failure if left untreated.

**Methods:**

This was an observational cross-sectional study conducted on 35 patients, treated for urethral stricture at the Department of Uro-nephrology Surgery, Ninh Thuan Province General Hospital, from January to October 2023.

**Results:**

All enrolled patients underwent urethral stricture endoscopic incision using holmium laser, and were followed up at 1 and 3 months postoperatively. The difference in the degree of hydronephrosis on CT scans before and after surgery at 3 months was statistically significant (*p* < 0.01).

**Discussion:**

To report the safety and efficacy outcomes of holmium laser urethrotomy for the treatment of urethral stricture, patients underwent internal urethrotomy with holmium laser energy, with an average age of 47.7 ± 15.8 years (range: 15-72 years). Thirty patients (85.7%) underwent urological surgery, 3 (8.6%) underwent obstetric and gynecological surgery, and 2 (5.7%) had unknown etiologic causes.

**Conclusion:**

The use of the holmium laser for the management of urethral strictures has been found to be safe and effective, ensuring shorter operating times, a lower recurrence rate, and fewer serious postoperative complications.

## 1 INTRODUCTION

Urethral stricture is a condition characterized by narrowing of the urethra that leads to functional changes, resulting in a reduction or complete obstruction of urinary flow from the kidneys to the bladder. It is a common urological disorder that has a significant impact on the quality of life of patients [1]. If left untreated, urethral stricture can cause urine retention at the narrow site, leading to hydronephrosis, kidney infection, and various complications, such as renal colic, stone formation, continuous urinary tract (UTI) infections, and eventually irreversible kidney damage [1, 2]. Cases of non-functional nephrectomy and abscess formation due to urethral stricture have also been recorded with a relatively high incidence rate (35.5%) compared to other causes [3].

Early diagnosis and treatment are of paramount importance. The development of modern imaging diagnostic methods, such as ultrasound, computed tomography scanning (CT scan), magnetic resonance imaging (MRI), and renal scintigraphy, has enabled an earlier and accurate diagnosis of the cause and severity of urethral stricture [4].

Therefore, the main goal of urethral stricture treatment is to relieve obstruction and preserve renal function. There are various procedures to treat urethral stricture, and reconstructive surgery is considered the gold standard, but it is a highly invasive procedure that can lead to numerous complications, prolonged hospital stays, and higher treatment costs [4, 5]. Surgery could be even life-threatening, particularly in patients with a history of multiple urinary surgeries, elderly patients, and multiple comorbidities. In recent years, the development of endoscopic techniques in the field of urology has led to effective and minimally invasive treatments for urethral strictures, achieving promising results [6]. Endoscopic urethral incision procedure is performed using a laser beam (low-power holmium laser - HO:YAG). This procedure is used to incise the urethral stricture, break the scar tissue, dilate the urethral lumen, and reduce hydronephrosis. This procedure has shown various advantages, including high success rates, ease of performance, low cost, and short hospital stays [7-9].

At Ninh Thuan General Hospital, endoscopic treatment of urethral stricture using the laser incision technique began to be implemented in early 2021, but it has not been standardized or made uniform. Evaluating treatment outcomes and documenting complications during surgery are crucial and surely improve the quality of treatment for patients. We performed holmium laser surgery using a 60-100-watt laser to create incisions and enucleate tissue, with a penetration depth of 0.4-0.5 mm, a key feature that allowed precise cutting with minimal damage to surrounding tissues. The incision range depends on the specific procedure and can vary; typically, the laser beam is used to make incisions for enucleation or resection of tissues. Based on this practical need, we conducted the current study to provide the necessary data to enable doctors to offer more treatment options for urethral stricture.

## 2 MATERIALS AND METHODS

### 2.1 Study Subjects

The patients with urethral strictures were diagnosed and treated at the Department of Urology and Nephrology, Ninh Thuan Provincial General Hospital, from January 2023 to October 2023.

### 2.2 Selection Criteria

Patients diagnosed with acquired urethral strictures, with a stricture length ≤2 cm, and a history of treatment for urethral conditions (surgery, internal medicine treatment for urinary stones), were included in the study.

### 2.3 Exclusion Criteria

Congenital urethral strictures, urethral cancer, invasive urethral cancers, and medical records lacking necessary information were excluded from the study.

### 2.4 Study Design

The study involved a cross-sectional case series design.

### 2.5 Sample Size and Sampling Method

The study involved 35 patients (29 males and 6 females), with a mean age of 47.7 ± 15.8 years (range 15-72 years).

### 2.6 Processing and Analyzing Data

The data were encoded and analyzed using SPSS software, employing the chi-square test and Fisher's exact test, with statistical significance considered at *p*<0.05.

### 2.7 Ethical Standards

The study ensured ethical standards through the following principles: no intervention was performed on study subjects, and all personal information and patient illustrations were kept confidential, processed, and published to ensure confidentiality.

The study was conducted after obtaining approval from the Scientific and Technical Council of Ninh Thuan Provincial Hospital. Informed consent was obtained from all the patients included in the study. The research procedures followed were in line with the ethical standards of the committee responsible for human experimentation (institutional and national), and with the Helsinki Declaration of 1975, as revised in 2013 (http://ethics.iit.edu/ecodes/node/3931; decision no. 735/QD-UBND, dated June 5, 2023, of the People's Committee of Ninh Thuan province; pursuant to decree no. 96/2023/ND-CP, dated December 30, 2023, with the government detailing a number of articles of the Law on Medical Examination and Treatment; pursuant to circular no. 43/2024/TT-BYT, dated December 12, 2024, of the Ministry of Health on the establishment, organization, and operation of the ethics council in biomedical research).

## 3 RESULTS

During the period from January 2023 to October 2023, there were 35 patients who met the criteria for inclusion in the study at the Department of Urology and Nephrology, Ninh Thuan Provincial Hospital. All 35 selected patients underwent urethral stricture endoscopic incision using holmium laser, and were followed up and evaluated for outcomes for at least 3 months postoperatively.

## 4 DISCUSSION

A urethral stricture involves damage that narrows the urethra, a condition commonly called stricture, limiting the exit of urine from the bladder [10-12]. This can cause problems, such as infections, inflammation, and damage to the entire genitourinary system, including the kidneys [11, 12]. The etiology of urethral strictures is generally linked to 4 main conditions: idiopathic, iatrogenic, inflammatory, and traumatic [13-15]. In our study, apart from idiopathic, traumatic, and iatrogenic causes, which are considered the most common, infection accounted for up to 26% of all treated patients. Strictures associated with infection tended to be relatively long, typically exceeding 4 cm [14-18].

Inflammatory strictures may result from post-infectious inflammation leading to weakening of the epithelium, known as recurrent gonococcal urethritis. These causes remain prevalent in countries with more limited resources [19-21]. Post-infectious urethritis is associated with recurrent UTI, with the most common organism being *Escherichia coli*. Although there is a possible genetic predisposition and an autoimmune factor, these types of urethral strictures only involve the anterior urethra and rarely involve the posterior urethra [20]. The main feature of these strictures is that they are much longer than those caused by other aetiologies and are more likely to require urethroplasty. Nevertheless, in cases of acute urinary retention or complications from urethral strictures or pathogens, urgent treatment may include various options, ranging from longer antibiotic treatment and urethral dilation to urethromy, cystoscopy, and even direct internal urethrotomy [22, 23]. These decisions, or rather, the decision itself, must always be coordinated and made to avoid further urethral trauma and discomfort for the patient. Our approach was to provide a period of urethral rehabilitation and healing rest before a more definitive procedure, such as urethroplasty [23-25].

In the present study, we confirmed the previously known risk factors for urethral stricture and the use of double-J stent, such as prolonged indwelling time, infection, inflammatory processes, and recurrent urolithiasis [26-28]. UPR procedure during surgery showed stricture mainly in the upper 1/3 part with 22 cases (56.8%), lower 1/3 with 11 cases (32.4%), and middle 1/3 with only 4 cases. (10.8%). The average urethral stricture length was 1.1± 0.6 cm (0.4-3 cm). Most narrow sections had a length of < 2 cm (accounting for 89.2%). No complications occurred during surgery in this study, accounting for 0%. The postoperative monitoring phase confirmed stable condition in 32 patients (91.4%), 2 cases of mild fever (38°C) on postoperative day 1 (5.7%), and 1 case of hematoma under the renal capsule and in the retroperitoneal area (2.9%). The results highlighted the importance of the Ho:YAG procedure not only from a clinical point of view, but also in identifying the most suitable patients to prevent complications, especially in subjects most at risk, such as those with diabetes and the elderly [29-31]. Ho:YAG lasers, like CO_2_ lasers, tend to provide a very clean cut, offering greater precision than CO_2_ without damaging surrounding tissue and the possibility of delivery *via* fiber optics (ideal for endoscopic use), allowing treatment of tissue in a fluid-filled environment, such as blood and saline [30, 31].

To our knowledge, this study is among the first in Vietnam to have thoroughly studied and clinically applied this laser technology and its potential implications.

### 4.1 Monitoring the Surgical Outcomes

The predominant clinical symptoms of the 35 patients with urethral lesions were mainly back pain (94.3%), chills (5.7%), and hematuria (2.9%). Among them, 1 patient experienced bilateral back pain, and 1 patient had both back pain and chills (Table **1**). Complete blood count (CBC) profile revealed that the majority of patients had white blood cell count within the normal range. Among 35 patients, 5 (14.3%) had white blood cell count above 10,000 cells/mm3, of whom 2 patients had symptoms of urinary tract infection, and they were appropriately treated with antibiotics. The white blood cell count returned to normal at the end of the antibiotic treatment when the infection subsided (Table **2**). Urea and creatinine analysis revealed an initial normal renal function for the majority of patients; 4 out of 35 patients showed elevated creatinine levels, 2 had a pre-existing condition of long-standing hypertension under continuous treatment, 1 was newly diagnosed with hypertension, and 1 had bilateral urethral stricture. Among these, 3 patients showed stage 3 chronic kidney disease (CKD), while 1 patient has been diagnosed with stage 4 CKD (Table **3**). Urinalysis showed that out of the total number, 18 patients had abnormal urine composition (53%), 6 showed asymptomatic white blood cells (1+), and 12 revealed elevated white blood cells (+++) (35%), of whom 5 were positive for nitrites. All patients with white blood cells in urine were subjected to urine culture, and the results revealed bacterial growth in 8 cases (*E. coli*) (24%) (Table **4**). Preoperative ultrasound and CT scan performed on all patients confirmed interesting results; the majority of the patients presented with hydronephrosis of grades 2 and 3 (89.2%) versus grade 1 (10.8%) (Tables **5** and **6**).

Four patients showed elevated creatinine levels, among whom two had a pre-existing condition of long-standing hypertension under continuous treatment, one was newly diagnosed with hypertension, and one had bilateral urethral stricture. Three patients were evaluated as having stage 3 chronic kidney disease, and one patient was classified as stage 4 chronic kidney disease.

There were 2 patients diagnosed with bilateral urethral stricture who underwent simultaneous endoscopic incision of the urethra (Table **7**). During intraoperative UPR imaging, urethral strictures were predominantly located in the upper third of the urethra in 22 cases (56.8%), followed by 11 cases (32.4%) in the lower third, and only 4 cases (10.8%) in the middle third. The average length of urethral stricture was 1.1 ± 0.6 cm (ranging from 0.4 to 3 cm). The majority of strictures had a length of less than 2 cm (accounting for 89.2%). The average surgical time was 48.7 ± 20.9 minutes (20-100 minutes). The majority of patients underwent surgery within < 60 minutes (65.7%) (Tables **8**-**10**). The difference in the degree of hydronephrosis on ultrasound and CT scan before and after surgery at 3 months was statistically significant (*p*<0.05 and *p* < 0.01) (Tables **11**-**13**).

At the one-month postoperative mark, all 34 patients underwent follow-up assessments to evaluate their clinical status, and the JJ stent was removed *via* cystoscopy. One patient was readmitted urgently due to significant hematuria events and high fever one month post-surgery. Despite internal medical treatment, the patient required removal of the JJ stent and underwent surgical reanastomosis of the urethra.

At 3 months after surgery, patients with renal pelvic dilation detected by ultrasound represented 65.6%; only 1 case presented an increase in dilation from grade 2 to grade 3. The remaining 34.4% did not present substantial changes. Therefore, the results of the clinical evaluation at three months after surgery were 31 in total, all of which showed positive results, while one urethra showed unfavorable results. This patient was confirmed to have a severe UTI associated with a reduction in renal function compared to the preoperative period (Table **14**).

Furthermore, we observed several factors related to the diagnosis of urethral stricture, with back hip pain being the most common clinical symptom (94.3%), and hydronephrosis detected by CT scan, accounting for the highest proportion (grade I: 8.1%; grade II: 48.6%; grade III: 43.3%). Endoscopic incision urethral stricture surgery demonstrated a successful treatment outcome, accounting for 78.3%, unaffected by the causes of stricture, degree of hydronephrosis, and stricture location; it exhibited a high safety profile with no recorded intraoperative complications and only 2 cases of mild postoperative complications (urinary tract infection accounted for 5.7% and subcapsular hematoma after nephrostomy accounted for 2.9%).

There was a correlation between the length of the urethral stricture and surgical outcomes, with statistically significant differences (*p* <0.05). Eventually, the endoscopic incision using holmium laser in urethral stricture treatment was safe and effective, with many advantages, such as short surgical time, short postoperative recovery time, and very few serious complications during and after surgery. However, randomized clinical studies with larger sample sizes and longer durations are needed to provide more detailed and accurate recommendations.

## CONCLUSION

The limitations of this study mainly included the small sample size, which may lead to risk of bias, limited generalizability, risk of random variability, lack of precision and reliability, and limited exploration of heterogeneity. However, with respect to possible adverse effects, this procedure has shown lower risks of general complications compared to other more conventional procedures. This methodology should be performed by taking into account individual conditions and patient preferences. Of course, future research with larger cohorts and extended follow-up periods is needed to refine further therapeutic recommendations.

## Figures and Tables

**Table 1:** Preoperative clinical symptoms.

Symptoms	Back Pain	Chills	Hematuria
Number of patients/urethra (35/37)	33	2	1
Percentage (%)	94.3	5.7	2.9

**Note:** The predominant clinical symptoms among 35 patients with urethral lesions were mainly back pain (94.3%), chills (5.7%), and hematuria (2.9%). Among them, 1 patient experienced bilateral back pain, and 1 patient had both back pain and chills.

**Table 2:** Preoperative white blood cell test results.

White Blood Cell Count/mm^3^	<4000	4000-10000	>10000	Total
Number of patients	0	30	5	35
Percentage (%)	0	85.7	14.3	100

**Note:** The majority of patients had white blood cell count within the normal range. Among 35 patients, 5 (14.3%) had white blood cell count above 10,000 cells/mm^3^, of whom 2 patients had symptoms of urinary tract infection, and they were appropriately treated with antibiotics. The white blood cell count returned to normal when the infection subsided.

**Table 3:** Preoperative biochemical test results.

Test		Normal	Increased	Total
Urea	Number of patients	33	2	35
	Percentage (%)	94.3	5.7	100
Creatinine	Number of patients	31	4	35
	Percentage (%)	88.6	11.4	100

**Note:** Urinary urea and creatinine tests were conducted to assess initial renal function, with the majority falling within the normal range (urea: 21-47 mg%, creatinine: 1-1.2 mg%).

**Table 4:** Preoperative urinalysis results.

Complete Urinalysis	Red Blood Cells	White Blood Cells	Nitrites
Number of patients	19	18	5
Percentage (%)	54.3	51.4	14.3

**Note:** The results of the complete urinalysis showed that out of 18 patients, 6 had a few white blood cells (1+) in their urine without symptoms of urinary tract infection. There were 12 patients with a high number of white blood cells in their urine, among whom 5 tested positive for nitrites. All patients with white blood cells in their urine underwent urine culture, and the results revealed bacterial growth in 8 cases.

**Table 5:** Preoperative ultrasound results.

Impact on the Kidney	Normal	Hydronephrosis			Total
		**Grade 1**	**Grade 2**	**Grade 3**	
Number of kidneys - urethra	0	4	24	9	37
Percentage (%)	0	10.8	64.9	24.3	100

**Note:** Preoperative ultrasound was performed on all patients. The results showed that hydronephrosis of grades 2 and 3 accounted for a high proportion of 89.2%. Grade 1 hydronephrosis accounted for 10.8%, and there were no cases without hydronephrosis.

**Table 6:** Preoperative CT scans of the urinary system.

Impact on the Kidney	Normal	Hydronephrosis				Total
		Grade 1	Grade 2	Grade 3	Grade 4	
Number of kidneys - urethra	0	3	18	16	0	37
Percentage (%)	0	8.1	48.6	43.3	0	100

**Note:** All 35 patients underwent CT scans of the urinary system to diagnose urethral stricture, and the results were consistent with the ultrasound findings regarding hydronephrosis. Hydronephrosis of grade 2 and grade 3 predominated, accounting for 91.9%. Grade 1 hydronephrosis was present in only 3 cases, constituting 8.1%, with no cases showing no hydronephrosis.

**Table 7:** Urethral side.

**Surgical Methods**	**Number of Urethra**	**Percentage (%)**
Bilateral surgery	4	10.8
Unilateral surgery (right or left)	33	89.2
Total	37	100

**Note:** There were 2 patients diagnosed with bilateral urethral stricture who underwent simultaneous endoscopic incision of the urethra.

**Table 8:** Location of urethral stricture.

Location of Stricture	1/3 Upper	1/3 Middle	1/3 Lower	Total
Number of urethral strictures	21	4	12	37
Percentage (%)	56.8	10.8	32.4	100

**Note:** During intraoperative UPR imaging, urethral strictures were predominantly located in the upper third of the urethra in 22 cases (56.8%), followed by 11 cases (32.4%) in the lower third, and only 4 cases (10.8%) in the middle third.

**Table 9:** he length of the urethral stricture was measured intraoperatively.

The Length of the Urethral Stricture (cm)	≤1	1.1-1.9	≥2	Total
Number of urethra	21	12	4	37
Percentage (%)	56.8	32.4	10.8	100

**Note:** The average length of urethral stricture was 1.1 ± 0.6 cm (ranging from 0.4 to 3 cm). The majority of strictures had a length of less than 2 cm (accounting for 89.2%).

**Table 10:** Surgery duration.

The Duration (in minutes)	Number of Patients	Percentage (%)
<60	23	65.7
60-90	11	31.4
>90	1	2.9
Total	35	100

**Note:** The average surgical time was 48.7 ± 20.9 minutes (20-100 minutes). The majority of patients underwent surgery within < 60 minutes (65.7%).

**Table 11:** Results of postoperative ultrasound at 3 months.

Renal Hydronephrosis Grading	Postoperative Ultrasound		Preoperative Ultrasound		**P**
	Number of Cases	Percentage (%)	Number of Cases	Percentage (%)	
No hydronephrosis	4	12.5	0	0	0.042
Grade 1	16	50	4	10.8	0.000
Grade 2	8	25	24	64.9	0.001
Grade 3	4	12.5	9	24.3	0.21
Total	32	100	37	100	-

**Note:** The difference in the degree of hydronephrosis on ultrasound before and after surgery at 3 months was statistically significant (*p*<0.05).

**Table 12:** The rate of reduction in the degree of hydronephrosis on ultrasound after surgery at 3 months.

Degree Conversion	Number of Cases	Percentage (%)
Yes	21	65.6
No	11	34.4
Total	32	100

**Note:** After 3 months post-operation, the percentage of renal pelvis dilatation changes on ultrasound accounted for 65.6%, with 1 case showing an increase in renal pelvis dilation (from grade 2 to grade 3). The remaining 34.4% of cases showed no change in renal pelvis dilation.

**Table 13:** Postoperative CT scan results at 3 months.

Degree of Hydronephrosis on CT Scans	Postoperatively		Preoperatively		**P**
No hydronephrosis	3	9.3	0	0	0.095
Grade 1	14	43.8	3	8.1	0.001
Grade 2	11	34.4	18	48.6	0.231
Grade 3	4	12.5	16	43.3	0.005
Total	32	100	37	100	-

**Note:** The difference in the degree of hydronephrosis on CT scans before and after surgery at 3 months was statistically significant (*p* < 0.01).

**Table 14:** The summary of factors used to evaluate surgical outcomes.

Factor	Quantity	Good	Average	Bad	Failed to Follow-up
Clinical	32	31	0	1	5
CTscan	32	20	11	1	

## Data Availability

All data generated or analyzed during this study are included in this published article.

## LIST OF ABBREVIATIONS

Ho-YAG: Holmium-doped Yttrium Aluminum Garnet
UTI: Urinary Tract Infection
JJ stent: Ureteral Stent Having Two Curled Ends
HPV: Human Papillomavirus
MRI: Magnetic Resonance Imaging
CT: Computed Tomography

## STANDARDS OF REPORTING

STROBE guidelines were followed.

## References

[1] Kairambayev Y., Bulegenov T., Omarov N., Kuderbayev M., Syzdykbayev M., Glushkova N., Akhmetzhanova D., Kaskabayeva A., Muzdubayeva Z., Akimzhanov K., Pivina L. (2024). Prevention of postoperative urethral strictures by irrigation with 5-fluorouracil *via* a modified urinary catheter.. Medicina (Kaunas).

[2] Raheem O.A., Buckley J.C. (2014). Adjunctive maneuvers to treat urethral stricture: A review of the world literature.. Transl. Androl. Urol..

[3] Roehrborn C.G., Barkin J., Gange S.N., Shore N.D., Giddens J.L., Bolton D.M.E., Cowan B.E., Cantwell A.L., McVary K.T., Te A.E., Gholami S.S., Moseley W.G., Chin P.T., Dowling W.T., Freedman S.J., Incze P.F., Coffield K.S., Herron S., Rashid P., Rukstalis D.B. (2017). Five year results of the prospective randomized controlled prostatic urethral L.I.F.T. study.. Can. J. Urol..

[4] (2019). Evaluation of outcomes of urinary tract infection treatment in patients with urinary tract stones after double J stent placement surgery.. Master's thesis in Medicine, University of Medicine and Pharmacy Ho Chi Minh City..

[5] Bauzá J.L., Calvó P., Julià F., Guimerà J., Martínez A.I., Tienza A., Costa-Bauzá A., Sanchís P., Grases F., Pieras E. (2023). Relationship between urinary parameters and double-j stent encrustation.. J. Clin. Med..

[6] Hibi H., Kato K., Mitsui K., Taki T., Yamada Y., Honda N., Fukatsu H. (2001). Endoscopic urethralal incision using the holmium: YAG laser.. Int. J. Urol.

[7] Hou C.P., Wu J.H., Weng S.C., Lin Y.H., Chen C.L., Tsai H.Y., Chen Y.T., Juang H.H. (2024). Urethral strictures after endoscopic enucleation of the prostate and its associated clinical outcomes in aging men.. Medicina (Kaunas).

[8] Tran Van Hinh (2013). Urethralal stenting.. Diagnosis and treatment methods for urinary stones..

[9] Van Ty V., Oanh D.Q., Thuan N.D. (2004). Endoscopic treatment of urethral stricture.. Ho Chi Minh City Med. J..

[10] Banner M.P., Pollack H.M., Ring E.J., Wein A.J. (1983). Catheter dilatation of benign ureteral strictures.. Radiology.

[11] Weiss D.A., Weiss R.M. (2020). Physiology and Pharmacology of the Renal Pelvis and Urethral..

[12] Abdeen B.M., Leslie S.W., Badreldin A.M. (2025). Urethral Strictures.. StatPearls..

[13] Santucci R.A., Joyce G.F., Wise M. (2007). Male urethral stricture disease.. J. Urol..

[14] Kochakarn W., Muangman V., Viseshsindh V., Ratana-Olarn K., Gojaseni P. (2001). Stricture of the male urethra: 29 years experience of 323 cases.. J. Med. Assoc. Thai..

[15] Bouassida K., Marzouk M., Nouir S., Ghammem R., Sahtout W., Ghardallou M., Fathallah N., Boukadida J., Jaidane M., Slim R., Zaïri A. (2023). Analysis of pathogens of urinary tract infections associated with indwelling double-J stents and their susceptibility to *Globularia alypum.*. Pharmaceutics.

[16] Scotland K.B., Lo J., Grgic T., Lange D. (2019). Ureteral stent-associated infection and sepsis: Pathogenesis and prevention: A review.. Biofouling.

[17] Kehinde E.O., Rotimi V.O., Al-Hunayan A., Abdul-Halim H., Boland F., Al-Awadi K.A. (2004). Bacteriology of urinary tract infection associated with indwelling J ureteral stents.. J. Endourol..

[18] Virasoro R., DeLong J.M., Estrella R.E., Pichardo M., Rodriguez Lay R., Espino G., Elliott S.P. (2022). A drug-coated balloon treatment for urethral stricture disease: Three-year results from the ROBUST I study.. Res. Rep. Urol..

[19] Whelan S., Lucey B., Finn K. (2023). Uropathogenic *Escherichia coli* (UPEC)-associated urinary tract infections: The molecular basis for challenges to effective treatment.. Microorganisms.

[20] Prakapaite R., Saab F., Planciuniene R., Petraitis V., Walsh T.J., Petraitiene R., Semoskaite R., Baneviciene R., Kalediene L., Kavaliauskas P. (2019). Molecular characterization of uropathogenic *Escherichia coli* reveals emergence of drug resistant O15, O22 and O25 Serogroups.. Medicina (Kaunas).

[21] Tulone G., Costanzo A., Pavan N., Giaimo R., Claps F., Fasciana T.M.A., Giammanco A., Bartoletti R., Simonato A. (2023). Analysis of bacterial stent colonization: The role of urine and device microbiological cultures.. Antibiotics.

[22] Lange D., Bidnur S., Hoag N., Chew B.H. (2015). Ureteral stent-associated complications—where we are and where we are going.. Nat. Rev. Urol..

[23] Wessells H., Morey A., Souter L., Rahimi L., Vanni A. (2023). Urethral stricture disease guideline amendment (2023).. J. Urol..

[24] Herout R., Flegar L., Putz J., Eisenmenger N., Huber J., Thomas C., Baunacke M. (2025). Increasing utilization of urethroplasty for male urethral stricture disease: Analysis of in-hospital interventions in Germany from 2006 to 2023.. Int. Urol. Nephrol..

[25] Tonyali S., Yilmaz M., Tzelves L., Emiliani E., De Coninck V.D., Keller E.X., Miernik A. (2023). Predictors of ureteral strictures after retrograde ureteroscopic treatment of impacted ureteral stones: A systematic literature review.. J. Clin. Med..

[26] Barakat B., Hadaschik B., Al-Nader M., Schakaki S. (2024). Factors contributing to early recovery of urinary continence following radical prostatectomy: A narrative review.. J. Clin. Med..

[27] Arsovska O., Paterson R,F., Chew B.H. (2015). Evaluation of risk factors and treatment options in patients with urethralic stricture disease at a single institution.. Can. Urol. Assoc. J..

[28] Hu H., Xu L., Wang S., Yu X., Yang H., Peng E., Cui L., Li C. (2017). Ureteral stricture formation after removal of proximal ureteral stone: retroperitoneal laparoscopic ureterolithotomy versus ureteroscopy with holmium: YAG laser lithotripsy.. PeerJ.

[29] Sánchez-Puy A., Bravo-Balado A., Diana P., Baboudjian M., Piana A., Girón I., Kanashiro A.K., Angerri O., Contreras P., Eisner B.H., Balañà J., Sánchez-Martín F.M., Millán F., Palou J., Emiliani E. (2022). New generation pulse modulation in Holmium:YAG Lasers: A systematic review of the literature and meta-analysis.. J. Clin. Med..

[30] Keller E.X., de Coninck V., Audouin M., Doizi S., Bazin D., Daudon M., Traxer O. (2019). Fragments and dust after Holmium laser lithotripsy with or without “Moses technology”: How are they different?. J. Biophotonics.

[31] Terry R.S., Ho D.S., Scialabba D.M., Whelan P.S., Qi R., Ketterman B.T., Preminger G.M., Zhong P., Lipkin M.E. (2022). Comparison of different pulse modulation modes for holmium:yttrium–aluminum–garnet laser lithotripsy ablation in a benchtop model.. J. Endourol..

